# Anchor Effect in Polymerization Kinetics: Case of Monofunctionalized POSS

**DOI:** 10.3390/polym11030515

**Published:** 2019-03-19

**Authors:** Agnieszka Marcinkowska, Dawid Przadka, Beata Dudziec, Katarzyna Szczesniak, Ewa Andrzejewska

**Affiliations:** 1 Faculty of Chemical Technology, Poznan University of Technology, Poznan, Berdychowo 4, 60-965 Poznan, Poland; daw-prza@wp.pl; 2 Faculty of Chemistry and Centre for Advanced Technologies, Adam Mickiewicz University in Poznan, Umultowska 89 B and C, 61-614 Poznan, Poland; beata.dudziec@gmail.com; 3 NanoBioMedical Center, Adam Mickiewicz University, Umultowska 85, 61-614 Poznan, Poland; k.szczesniak@amu.edu.pl

**Keywords:** Monomethacryloxy POSS, lauryl methacrylate, polymerization kinetics, anchor effect

## Abstract

The effect of the anchoring group on the detailed polymerization kinetics was investigated using monomethacryloxy-heptaisobutyl POSS (1M-POSS). This compound was copolymerized with lauryl methacrylate (LM) as the base monomer, at various molar ratios. The process was initiated photochemically. The polymerization kinetics were followed by photo-DSC and photorheology while the polymers were characterized by nuclear magnetic resonance (NMR), gel permeation chromatography (GPC), transmission electron microscopy (TEM), and differential scanning calorimetry (DSC). For comparison, a methacrylate containing the branched siloxy-silane group (TSM) was also studied. It was found that the modifiers with a bulky substituent have a dual effect on the termination process: (i) At low concentrations, they increase the molecular mobility by increasing the free volume fraction, which leads to an acceleration of the termination and slows the polymerization; while (ii) at higher concentrations, they retard molecular motions due to the “anchor effect” that suppresses the termination, leading to acceleration of the polymerization. The anchor effect can also be considered from a different point of view: The possibility of anchoring a monomer with a long substituent (LM) around the POSS cage, which can further enhance propagation. These conclusions were derived based on kinetic results, determination of polymerization rate coefficients, and copolymer analysis.

## 1. Introduction

In recent years, there has been growing interest in high-performance inorganic–organic hybrid polymer materials. Both components (the inorganic modifier and the polymer matrix) can be linked together physically or by chemical links. Good compatibility between the components can be provided by functionalization of the inorganic filler with organic substituents. A widely studied novel class of nanofillers, in which the inorganic core can be functionalized by a wide range of organic substituents, are polyhedral oligomeric silsesquioxanes (POSSs). POSSs are organic–inorganic hybrid compounds constituted by an inorganic silica cage described by the general formula Rn(SiO_1.5_)_n_ or *T_n_*, where n is the number of repeat units (n = 8, 10, or 12, *T* is RSiO_1.5_) and R is H or an organic substituent. This results in the POSS molecule having a unique shape: the inorganic core is surrounded by an organic outer layer. The size of the POSS cage is ~1.5 nm [1].

The substituents, R, can be reactive (e.g., containing double bonds, epoxy groups) or nonreactive, of any structure. These attached functional groups improve the solubility/compatibility of POSS with polymers and lead to the improvement of many properties, such as the thermal stability, thermomechanical and electrical properties, flame retardancy as well as mechanical strength [2,3,4,5]. POSS cages are considered as the smallest possible form of silica [5]. POSS compounds can be soluble in the polymer matrix; therefore, they can be dispersed on the molecular level, which enables them to control the motions of the chains and to control the physical properties of the base polymer. In such a case, true hybrid materials are formed. However, POSS molecules or POSS moieties have a tendency to agglomerate (or even form crystallites), which in turn leads to the formation of (nano)composites. POSSs can be introduced into the polymer matrix by physical blending or via copolymerization (covalent bonding into a polymer backbone) [6].

Copolymerization is the most common approach used to obtain polymer/POSSs materials. When POSS contains only one reactive substituent, it can be incorporated into the polymer as a pendant unit; if it bears more reactive groups, it will form cross-linking points in the arising network. Widely investigated systems are methacryloxy-based POSS-containing materials. Copolymerization of various methacrylates with methacryloxy-substituted POSS has been described in a number of papers. Both thermal and photochemical initiation were applied [7].

UV-curable polymer systems are of particular interest in the development of new materials. This technology offers a number of advantages, namely, ultrafast curing, ambient temperature operation, and spatial and temporal control of the process. Additionally, it has an environment-friendly aspect (no emission of volatile organic compounds, low energy consumption). UV-curing has many important modern applications, e.g., in the coatings industry, dentistry, lithography and microlithography, microelectronics, optoelectronics, etc. [7,8].

Existing articles have been devoted to various aspects of the POSS effect, mainly to the improvement of mechanical and thermal properties as well as to morphology and, in general, to structure–property relationships. However, it is very important to know the polymerization kinetics, because they determine, to a large extent, the properties of the material, provide information on the optimal monomer/modifier ratios, and enable the selection of the technological parameters of curing.

Despite many publications being devoted to methacryloxy-based POSS-containing materials, the number discussing the influence of POSS compounds on the polymerization kinetics is very limited. There is a lack of deeper insight into the kinetics reaction; only a general discussion was presented in a few reports, e.g., [9,10,11,12,13,14,15,16] both using monomethacryloxy (1M, linear monomer) as well as octamethacryloxy (8M, a crosslinking monomer) POSS. Mainly the effect of the addition of functionalized POSS on the polymerization rate and double bond conversion of methacrylate-based formulations was studied, but the results and possible explanations differed; e.g., a decrease in the final conversion (both with 8M- and 1M-POSS, explained by the steric hindrance and the reduction of the polymer network mobility associated with the inorganic part of these POSSs) [9], reduction of both the double bond conversion and the polymerization rate (with 8M-POSS) [11], acceleration and enhanced conversion or slowdown and reduced conversion, depending on the reaction stage (due to both an enhancement of the gel effect and blocking light absorption by the photoinitiator, octavinyl-POSS) [12], and an increase in the double bond conversion and polymerization rate due to an increase in the concentration of double bonds (8M-POSS [15] and 1M-POSS [16]). The more detailed study describing the effect of 8M-POSS on the formation of a rigid polymer (poly(2-hydroxyethyl methacrylate)) or an elastomer (poly (oxyethylene glycol (di)methacrylate)) in a wide range of its concentration was presented in [7]. The final double bond conversion decreased with the increase of the POSS content, but the polymerization rate first increased and then decreased, reaching its highest value in the presence of about 15–25 wt % of the modifier (depending on the monomer). These phenomena were related to the influence of the POSS cage and the polymer cross-linking on the diffusional ability of the reacting species and resulting changes in the termination mechanisms.

However, it is obvious that when 8M-POSS is used, its crosslinking effect will dominate the polymerization kinetics and the effect of the POSS cage will be much less pronounced. Therefore, the best model POSS monomer would be a monofunctionalized compound, which, after incorporation into the polymer, will form a linear copolymer.

It was indicated that POSS moiety, incorporated into the polymer chain as a massive and bulky pendant group, serves as an anchor point bound to the chain. The presence of POSS moieties (nondiffusive “anchors”) along the backbone of polymer chains may dramatically alter the diffusion of polymer chains and slow down molecular motions [17,18,19]. This, in turn, can also have a significant effect on the polymerization kinetics.

**Figure 1:**
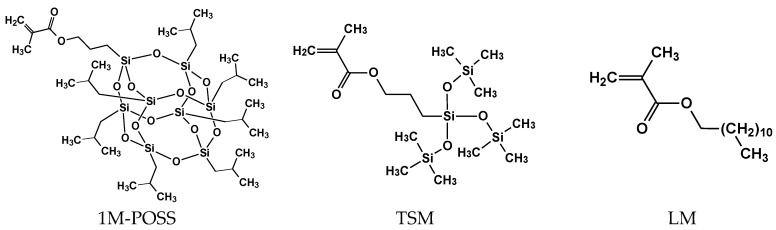
Structures of the monomers used.

The aim of the work was to investigate the possible anchoring effect exerted by the POSS cage on the polymerization kinetics of the UV-initiated process. The model system consisted of a monomethacryloxy substituted POSS (methacryloxypropyl-heptaisobutyl POSS, denoted as 1M-POSS) and lauryl methacrylate (LM, the only popular and non-volatile methacrylate monomer miscible with 1M-POSS) at various comonomers ratios. The investigated system is linear; therefore, its polymerization kinetics will not be complicated by crosslinking effects. For comparison, also another methacrylate having a large volume substituent (corresponding to an open part of the POSS cage, which may also serve as an anchor) was investigated, namely 3-[tris(trimethylsiloxy)silyl]propyl methacrylate (TSM) (Figure 1). This provides better insight into the possible “anchor effect”. The polymerization was initiated photochemically, which enables precise control of the reaction (the initiator derived radicals begin to form when the light is switched on and their formation is stopped at the break of irradiation; the polymerization occurs only in the irradiated area [20]).

## 2. Materials and Methods

### 2.1. Materials

TSM (purity 98%) and LM (purity 96%) were delivered by Sigma-Aldrich; they were purified by column chromatography (aluminiumoxid 90 Activ basisch, Merck, Darmstadt, Germany) before use and were stored over molecular sieves. The photoinitiator, 2,2-dimethoxy-2-phenylacetophenone, was also supplied by Sigma-Aldrich and was used in conc. 1 wt %.

#### 1M-POSS Preparation

1M-POSS (full name: 1-methacryloxypropyl-3,5,7,9,11,13,15-hepta(isobutyl)pentacyclo-[9.5.1.13,9.15,15.17,13] octasiloxane) was prepared in a two-step procedure, i.e., condensation of 1,3,5,7,9,11,14-heptaisobutyltricyclo [7.3.3.15,11] heptasiloxane-endo-3,7,14-triol with trichlorosilane, resulting in 1-hydro-3,5,7,9,11,13,15-hepta(isobutyl)pentacyclo [9.5.1.13,9.15,15.17,13] octasiloxane and its subsequent hydrosilylation with allyl methylacrylate using a Karstedt catalyst [21]. All solvents and liquid reagents were purchased from Merck and ABCR (Karlsruhe, Germany), and were dried and distilled under argon prior to use. The structure of the obtained compound was confirmed by spectroscopic analysis [22]. ^1^H, ^13^C, ^29^Si NMR spectra were recorded on Bruker Avance 300 MHz and 400 MHz (Bruker Scientific LLC, Billerica, MA, USA) in CDCl_3_.

^1^H NMR (CDCl_3_, 400 MHz, δ, ppm): 0.59–62 (m, 16H, SiCH_2_), 0.95 (dd, 42H, CH_3_), 1.73–1.79 (m, 2H, SiCH_2_CH_2_), 1.81–1.89 (m, 7H, CH_2_CHCH_3_), 1.95 (s, 3H, CH_3_), 4.11 (t, JH-H = 6.6Hz, 2H, CH_2_CH_2_CO), 5.55, 6.11 (s, 2H, CH_2_=).

^13^C NMR (CDCl_3_, 100 MHz, δ, ppm): 8.88 (SiCH_2_), 18.79 (CH_2_CH_2_), 22.98 (CH_2_CH(CH_3_)_2_), 24.33 (CH_2_CH(CH_3_)_2_), 26.16 (CH_2_CH(CH_3_)_2_), 67.07 (CH_2_O), 125.63 (=CH_2_), 137.01 (=CCH_3_), 167.94 (C=O). ^29^Si NMR (CDCl_3_, 79 MHz, δ, ppm): −67.70, −68.56, −68.85 (SiO_3_).

### 2.2. Methods

#### 2.2.1. Viscosity

The viscosity of the photocurable systems was measured with a DV-II+ PRO Brookfield Digital Viscometer (Brookfield, Toronto, Ontario, Canada) at the polymerization temperature.

#### 2.2.2. Glass Transition

Glass transition temperature, *T_g_*, was measured with a Mettler Toledo DSC1 instrument (Mettler Toledo GmbH, Schwerzenbach, Switzerland) under a nitrogen atmosphere at a heating rate of 20 °C/min in the temperature range from −80 °C to 180 °C. The *T_g_* value was determined from the second run of the DSC measurement and was taken as an average value from three measurements. The reproducibility of the results was about ±4%.

#### 2.2.3. NMR Study

^1^H and ^13^C NMR spectra were performed on Bruker Ultra Shield 400 and 300 spectrometers (Bruker Scientific LLC, Billerica, MA, USA) using toluene-d_8_ and CDCl_3_ as solvents. Chemical shifts are reported in ppm with reference to the residual solvents’ (C_7_H_8_, CHCl_3_) peaks for ^1^H and ^13^C NMR.

#### 2.2.4. Photopolymerization Kinetics

Reaction rate profiles were monitored by DSC using a Pyris 6 instrument (Perkin–Elmer, Waltham, MA, USA) equipped with a lid especially designed for photochemical measurements. The 15 mg samples were polymerized in open aluminum pans (diameter 6.6 mm) under isothermal conditions (40 °C) in high-purity argon atmosphere (<0.0005% of O_2_). The polymerization was initiated by the UV light (365 nm, light intensity 2 mW·cm^−2^) from the LED lamp (LC-L1, Hamamatsu Photonics, Hamamatsu, Japan). All photopolymerization experiments were conducted at least in triplicate. The reproducibility of the kinetic results was about ±3%. For computations, the heat of the polymerization of the methacrylate group, 56 kJ·mol^−1^, was taken [23].

The experimental data for the calculations of the polymerization rate coefficients: Propagation rate coefficient (*k_p_*) and bimolecular termination rate coefficient (*k_t_^b^*) in the expression for the polymerization rate (Equation (1)) [20]:

(1) $$R_{p}=\frac{k_{p}}{{\left(k_{t}^{b}\right)}^{0.5}}\cdot \left[M\right]\cdot {\left(\phi \cdot I_{a}\right)}^{0.5}$$

where [*M*] is the double bond concentration, *ϕ* denotes the quantum yield of initiation, and *I_a_* is intensity of the light absorbed, and were obtained from postpolymerization processes (non-steady-state measurements), which were registered after stopping the irradiation at various degrees of the double bond conversion. The calculations were performed over the first 10 s of the dark reaction. The rate coefficients were determined according to the bimolecular termination model [20]:

(2) $$\frac{{\left[M\right]}_{t}}{{\left(R_{p}\right)}_{t}}=\frac{2\cdot k_{t}^{b}}{k_{p}}\cdot t+\frac{{\left[M\right]}_{0}}{{\left(R_{p}\right)}_{0}}$$

where (*R_p_*)*_t_*, (*R_p_*)_0_ are the polymerization rates at time, *t*, of the dark reaction and at the moment of breaking the irradiation, resp., and [*M*]*_t_* and [*M*]_0_ are the concentrations of double bonds after the time, *t*, of the dark reaction and at the moment of breaking the irradiation. Equation (2) allows the determination of the *k_t_^b^/k_p_* ratio. Using Equation (2) (non-stationary conditions) and Equation (3) (from steady-state measurements):

(3) $${\left(R_{p}\right)}_{0}=\frac{k_{p}}{{\left(k_{t}^{b}\right)}^{0,5}}\cdot {\left[M\right]}_{0}\cdot {\left(F\right)}^{0,5}$$

where *F* = *ϕ·I_a_*, we can determine the polymerization rate coefficients in the form of *k_p_ × F* and *k_t_^b^ × F*. It was assumed that *F* is constant in the range of the conversions studied. A detailed procedure of the calculations was described in [20,24].

It should be emphasized that the measured parameters concern the copolymerization process as a whole; the double bond conversion involves the conversion of both comonomers while the polymerization rate is the overall rate of the C=C conversion of the two comonomers.

#### 2.2.5. Photorheology

Real-time photorheology experiments were performed on an Anton Paar MCR 301 rheometer (Anton Paar, Graz, Austria) using parallel plate geometry of a quartz plate and metallic plate (disposable D-PP25-SN0 measuring system, Anton Paar, Graz, Austria) with a diameter of 25 mm and a gap thickness of 0.2 mm. The measurements were carried out at 40 °C (in the heating chamber); the rheometer operated at a frequency of 10 Hz (oscillatory mode). Polymerization was initiated 30 s after starting the measurement by switching on the UV light (320–500 nm, 1 mW∙cm^−2^ from an OmniCure S 1000 high-pressure mercury lamp (OmniCure, London, England) projected via a waveguide into the sample through the bottom quartz window. During the measurements, the change in the normal force along with the storage modulus, G’, and loss modulus, G”, were recorded. The measurement for each sample was run for 2400 s.

#### 2.2.6. GPC

GPC analyses were performed using Agilent 1260 Infinity system (Agilent Technologies, Santa Clara, Utah, USA) equipped with RI detector and Phenogel 10 μm Linear(2) 300 × 7.8 mm column. THF was used as a mobile phase in a flow rate of 1.0 mL·min^−1^. Temperatures of RI detector and column were set at 35 °C. Time of analysis was 15 min. Molecular weights (number average, *M_n_*; weight average, *M_w_*) and polydispersity index (PDI) values were calculated based on the calibration curve using polystyrene standards (Shodex) in a range of 1000–3,500,000, using Agilent Software GPC/SEC – 1260 GPC set.

#### 2.2.7. TEM

TEM images were recorded on a JEM-1400 microscope made by JEOL (Tokyo, Japan). The accelerating voltage was 120 kV. A small amount of the samples was applied on a copper grid (Formvar/Carbon 200 mesh made by TedPella Inc., Redding, CA, USA).

## 3. Results and Discussion

The photopolymerization kinetics of the LM/1M-POSS and LM/TSM systems were followed at 40 °C in a wide range of the comonomer ratios. The kinetics were investigated both in general terms (kinetic curves) as well as by determination of the polymerization rate coefficients. Due to the fact that the effects of two different monomers with bulky substituents were compared, the concentrations of the formulation components were set in mole percent. 1M-POSS showed a limited solubility with LM (up to 12 mol %), whereas TSM was soluble in every ratio; therefore, the range of the investigated (base monomer)/modifier compositions was determined by the limits of miscibility. The composition of the investigated formulations (given in mol % and wt %) is given Table 1. 1M-POSS is a solid (m.p. 160 °C from DSC), therefore, its homopolymerization could not be followed.

### 3.1. Viscosity of Formulations

The initial viscosity of a polymerizable composition (along with that growing during the polymerization) is the important factor influencing the termination rate coefficient (an inverse proportionality: *k_t_^b^~η^−1^*). Thus, it is necessary to consider its changes when the ratio of the comonomers is changed.

**Figure 2:**
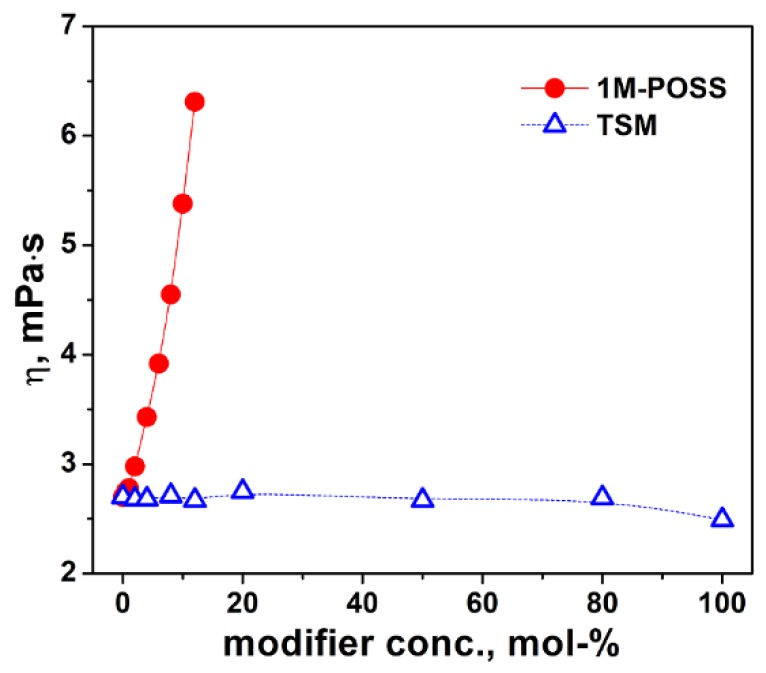
Viscosity of LM/1M-POSS and LM/TSM systems at 40 °C as a function of the Si-containing modifier concentration. The lines are eye guides.

The dependence of the viscosity on the modifier content for the two systems (LM/1M-POSS and LM/TSM) is given in Figure 2. The viscosity of the LM/1M-POSS system rapidly increases with the 1M-POSS content, indicating the existence of strong interactions between the POSS-cages (not yet tethered to the polymer chain) and the LM monomer.

On the other hand, the viscosity of the LM/TSM system is low and changes very slightly (LM: *ƞ*^40^ = 2.7 mPa·s, TSM: *ƞ*^40^ = 2.49 mPa·s). This proves changes in viscosity due to changes in the monomer ratio will practically not affect the polymerization.

### 3.2. NMR Analysis

**Figure 3:**
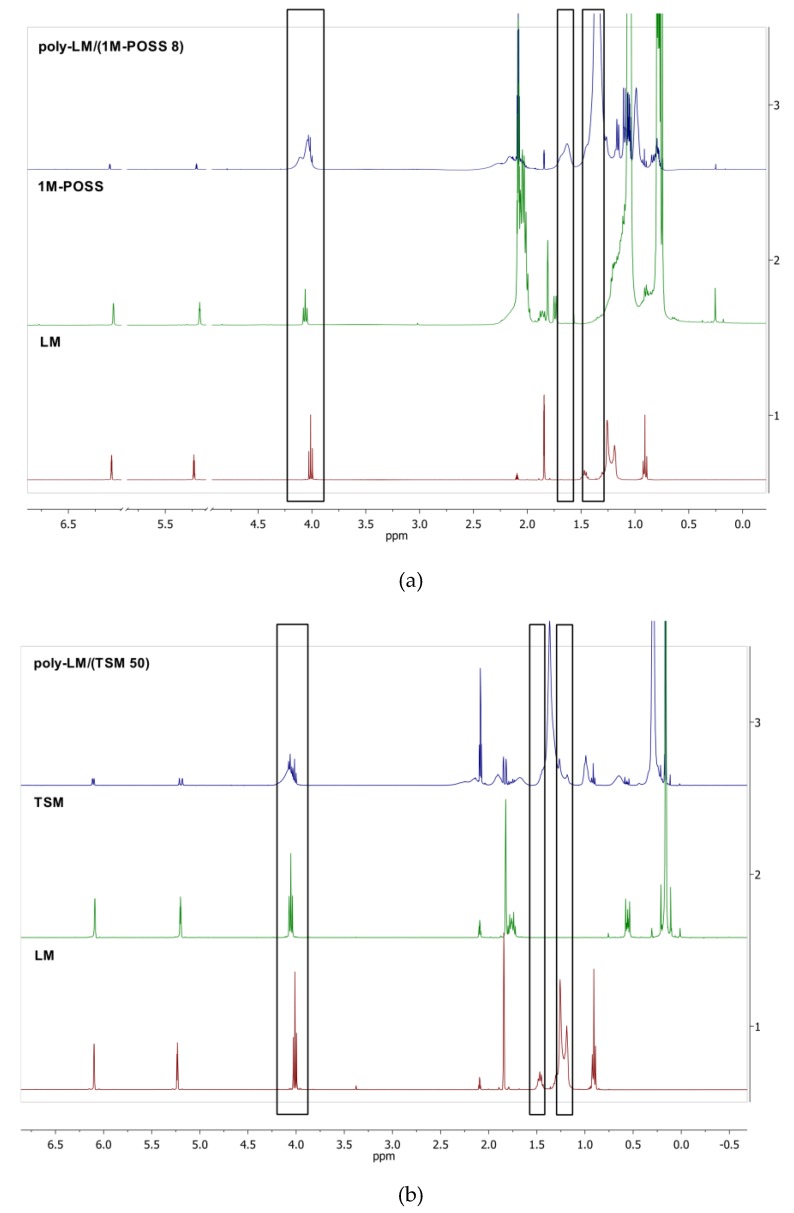
^1^H NMR stacked spectra of the LM monomer, the modifiers, and the copolymers containing (**a**) 8 mol % of 1-M POSS, (**b**) 50 mol % of TSM.

**Figure 4:**
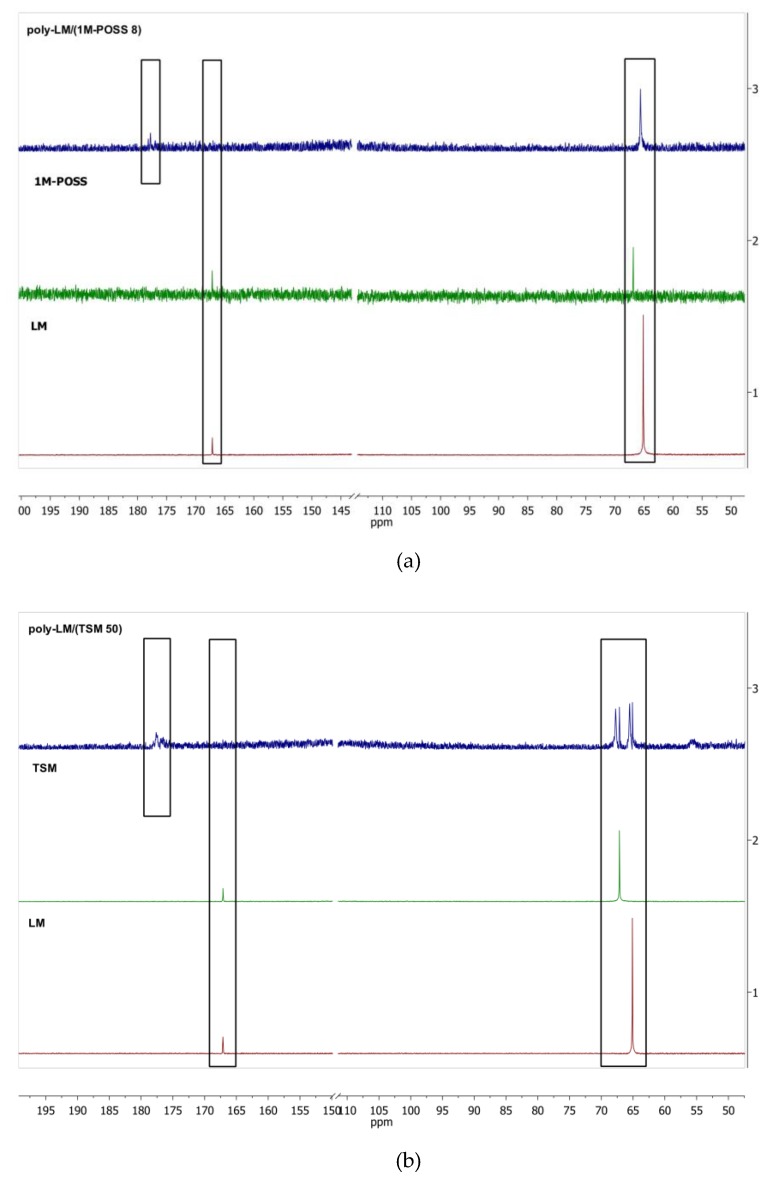
^13^C NMR stacked spectra of the LM monomer, the modifiers, and the copolymers containing (**a**) 8 mol % of 1-M POSS, (**b**) 50 mol % of TSM.

^1^H and ^13^C NMR spectra of the monomers and (co)polymers containing 8 mol % of 1M-POSS or 50 mol % of TSM are shown in Figure 3 and Figure 4, respectively.

In Figure 3, the ^1^H NMR stacked spectra disclose the correlations between the ^1^H NMR spectra of LM, 1M-POSS, and their copolymer (Figure 3a) and LM, TSM, and their copolymer (Figure 3b). The resonance lines originating to specific types of groups of both comonomers are visible. There are new signals arising from the -CH_2_- (polymer chain) at 1.63 ppm (wide resonance line) and 1.35 ppm, along with the changes in the region between 4.10–4.00 ppm that is assigned to the -OCH_2_- groups deriving from the copolymer and monomers. The surrounding -OCH_2_- chemical is slightly changed in the fragments derived from LM and 1M-POSS. This region is the most susceptible to changes and they are obviously more noticeable with the rising 1M-POSS content in the copolymer. In the case of the TSM-containing copolymer, there is a resemblance in the matter of the -OCH_2_- moiety and new signals arising from the -CH_2_- fragments.

The ^13^C NMR spectra confirm the appearance of new resonance lines at ca. 177.3 ppm that can be attributed to the carbonyl moiety (C=O) in the newly formed copolymer and this region is shifted towards higher values of ppm when compared to the LM monomer and 1M-POSS (166.7 ppm) (Figure 4a). In addition, there are also slight changes of signals in the region of 64-66 ppm originating from -OCH_2_- groups and a new signal appears at 65.16 ppm, which can be attributed to the copolymer, while the LM and 1M-POSS monomers show 64.6 and 66.4 ppm resonance lines, respectively. An analogous comparison was made for LM, TSM, and poly-LM/(TSM 50) (Figure 4b).

There is a similarity with regard to the new resonance lines at about 177.49 ppm derived from carbonyl groups (C=O), as well as the region corresponding to the -OCH_2_- moiety, i.e., the presence of new peaks at 67.75 and 65.53 ppm from the poly-LM/(TSM 50) copolymer together with residual signals at 67.15 and 65.11 ppm assigned to the LM and TSM monomers.

The ^1^H NMR spectra of the copolymers reveal the presence of chemical shifts (as residual resonance lines) of the methylene protons at 6.11 and 5.21 ppm derived from the double bonds of the monomers.

From the spectra of the (co)polymers with different modifier contents, the conversions of the monomers were calculated by an evaluation of the integration ratio of methylene protons with the rest of the protons’ contribution before and the after (co)polymerization. The results are given in Table S1. They are discussed in Section 3.4.1.

### 3.3. Photopolymerization Kinetics

#### 3.3.1. DSC Study

**Figure 5:**
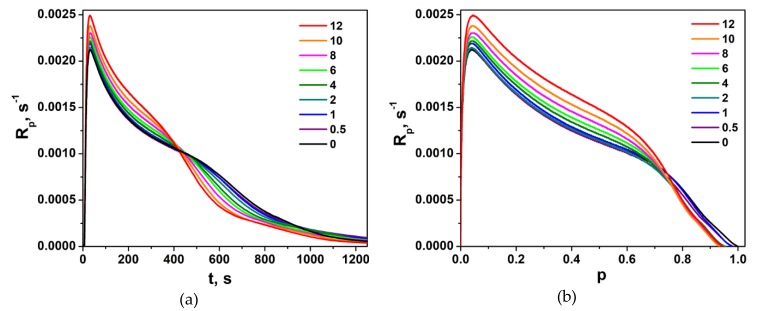
Polymerization rate, *R_p_*, of LM/1M-POSS compositions as a function of (**a**) the irradiation time, *t*, and (**b**) double bond conversion, *p*, at 40 °C. The numbers indicate the 1M-POSS content (mol %) in the mixture.

**Figure 6:**
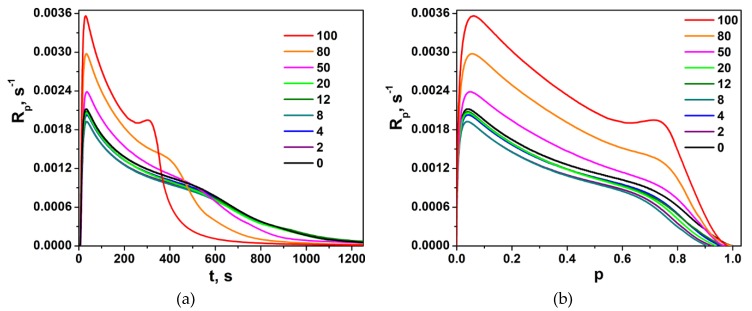
Polymerization rate, *R_p_*, of LM/TSM compositions as a function of (**a**) the irradiation time, *t*, and (**b**) double bond conversion, *p*, at 40 °C. The numbers indicate the TSM content (mol %) in the mixture.

Polymerization traces of neat LM and LM/1M-POSS mixtures, expressed as the dependence of the polymerization rate, *R_p_*, on the irradiation time, *t*, and on the degree of the double bond conversion, *p*, are shown in Figure 5. The corresponding curves for the LM/TSM formulations are presented in Figure 6.

The homopolymerization of neat LM (as other methacrylates with a longer ester group) occurs, with a poorly marked gel effect appearing at higher conversions [25]). Consequently, the polymerization rate at the maximum of this effect is much lower than at the beginning of the polymerization, in contrast to short-chain methacrylates, such as methyl methacrylate MMA (e.g., [26]), butyl methacrylate, or 2-hydroxyethyl methacrylate [7]. Therefore, in our case, the maximum polymerization rate, *R_p_^max^*, is defined as the highest reaction rate, not the rate at the maximum of the gel effect.

TSM homopolymerizes faster than LM, and the gel effect is more visible (its maximum occurs at about *p* ~ 0.7 ÷ 0.8 (Figure 6). As mentioned above, homopolymerization of 1M-POSS in bulk could not be followed because this compound is solid at 40 °C.

Figure 5 and Figure 6 suggest that the introduction of both modifiers into the base monomer increases the polymerization rate. Usually, the first explanation that comes to mind (and is most often right) is the reduction of the termination rate. This can result both from the increase in the initial viscosity of the formulation, as well as from the expected effect of the bulky substituent affecting the diffusion. These both factors can influence the polymerization of 1M-POSS-containing compositions, but the effect of TSM must result only from the bulkiness of the substituent (no influence of the formulation viscosity). It was speculated, for example, that much faster polymerization of 1-adamantyl methacrylate (AdMA) than other methacrylates (MMA, tert-butyl methacrylate, cyclohexyl methacrylate) is caused by the reduction of *k_t_* (not determined in the paper cited) due to the steric effect of the substituents, i.e., the mobility of the segment around the radical center [27]. In this case, the adamantyl group acts as an anchor and sterically blocks termination, which results in the increased concentration of propagating radicals.

The second possibility of the acceleration by the modifiers is an increase in the propagation rate coefficient, *k_p_*, and this will be discussed in Section 3.3.3. Such a possibility was rather excluded in the case of AdMA [27].

**Figure 7:**
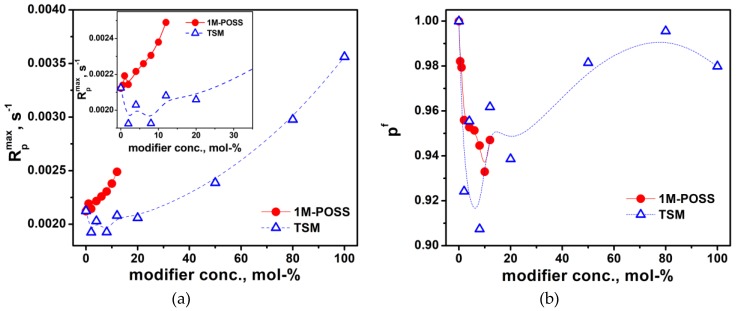
The dependence of (**a**) the maximum polymerization rate, *R_p_^max^*, and (**b**) the final conversion of double bonds, *p^f^*, at 40 °C on the modifier content. The lines are eye guides.

A more detailed analysis of Figure 5 and Figure 6 indicates, however, that the increase in the polymerization rate in the presence of the modifiers does not occur in the whole range of their concentrations. Figure 7a presents *R_p_^max^* values as functions of the content of the Si-containing monomers. It is clearly visible that the addition of their small amounts slows down the reaction. This effect is well observable in the case of LM/TSM compositions; the lowest *R_p_^max^* value appears at a TSM concentration about 8 mol %; at higher concentrations, the polymerization rate begins to increase. For the LM/1M-POSS system, the clear minimum is not visible due to the scattering of the data and only a kind of a plateau up to 2 mol % of 1M-POSS content can be observed (the minimum appears at 1 mol % of the 1M-POSS content as can be found from rheological studies, see Section 3.3.2). This tendency is reflected also by the behavior of the final conversion of double bonds, *p^f^*, Figure 7b (the determination of *p^f^* is always subject to a greater error, hence the considerable scatter of points). Further discussion of these phenomena first requires consideration of the behavior of the gel effect.

**Figure 8:**
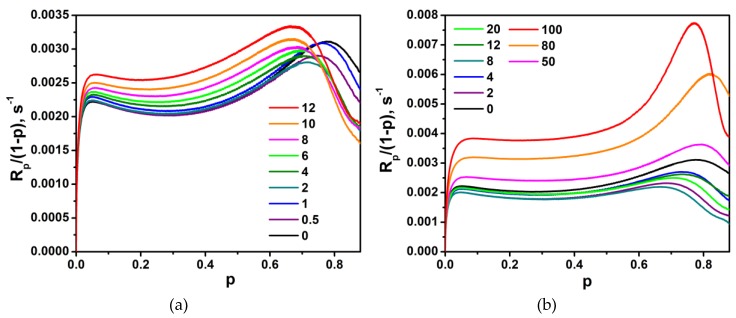
*R_p_/(1-p)* as a function of the double bond conversion, *p*, at 40 °C for (**a**) LM/1M-POSS and (**b**) LM/TSM systems. The numbers indicate the modifier content (mol %) in the composition.

As mentioned above, the gel effect in LM-based formulations is poorly marked. We can observe it better on the plots showing the dependence of the concentration of radicals on the conversion degree. The polymerization rate is expressed by Equation (4):

(4) $$R_{p}=k_{p}\left[M\right]\left[M^{\cdot }\right]=k_{p}\left[M^{\cdot }\right]\left(1-p\right)$$

where *[M^.^]* is the concentration of radicals, and *k_p_* is a constant up to high conversions. Therefore, the corresponding changes in the radical concentrations during the polymerization can be expressed readily by the function *R_p_/(1−p) = f(p)* (Figure 8). As could be expected, for the LM/1M-POSS system, the beginning of the gel effect (the beginning of the increase in the *R_p_/(1−p)* value) as well as the maximum of the effect shift to lower conversions with an increasing modifier concentration, which can result to a high degree from the increase in the viscosity of the formulations.

However, the addition of small amounts of 1M-POSS (<~4 mol %) at first weakens the gel effect (a decrease in the value of the *R_p_*/(1 − *p*) function); the enhancement is observed above this concentration. An analogous phenomenon, even more pronounced, occurs in the case of the LM/TSM system. The weakening of the gel effect, which in this case is also associated with the decrease in the polymerization rate and decrease in the radical concentration in the steady-state region, occurs for compositions containing up to at least 12 mol %; above this concentration, an enhancement of the gel effect is observed.

The results presented show that the effect of adding a comonomer containing an anchor group to the base monomer is twofold: At low comonomer concentrations, it leads to a decrease in the reaction rate, but after exceeding a certain concentration threshold, the trend is reversed and the polymerization rate begins to increase, reaching, at higher modifier contents, significantly higher values compared to the polymerization of the base monomer.

The weakening of the gel effect indicates an enhanced mobility of the radical centers (both by segmental and translational diffusion of macroradicals), which results in the acceleration of termination and leads to the drop of *R_p_*. In the case of TSM-containing formulations, this slowing down effect is stronger than in the case of 1M-POSS probably due to practically no change in the initial viscosity of the formulations. For the LM/1M-POSS system, the enhancement of macroradical mobility by the introduction of 1M-POSS moieties overcomes the effect of the increased viscosity, which slows down the translational diffusion and accelerates the reaction.

It has been suggested that introduction of methacryloxypropyl-heptaisobutyl POSS into the polymer chain can increase molecular mobility by increasing the free volume fraction through the bulky substituent and the plasticizing effect exerted by isobutyl groups [19,28]. We can assume that analogous effects are responsible for the kinetic behavior of the formulations containing small amounts of both modifiers. On the other hand, it is often indicated that interchain interactions between the massive inorganic POSS cages (“anchor objects”) [19] incorporated as pendant groups are responsible for the retardation and slow down of molecular motions [18,29]. This effect, which suppresses the termination, may in turn be one of the reasons for the acceleration of the polymerization in the presence of a larger amount of the modifiers. After exceeding a threshold concentration (about 2–4 mol %), the “anchor effect” begins to overcome the previously dominant effect of the increasing chain mobility. Therefore, minima appear on the dependencies, *R_p_^max^ = f(modifier conc.)* and *p^f^ = f(modifier conc.)*. The threshold concentration is higher for TSM due to the smaller “anchor group” and lower viscosity. However, it should be remembered that the anchor effect can only be one of the reasons for the increase in the polymerization rate.

An additional complicating, but important parameter is the reactivity ratio of the two comonomers. From the data provided by hybrid plastics [30,31], in a mixture of methyl methacrylate (MMA) and monomethacryl-based POSS, the POSS reactivity ratio, *r_POSS_*, is below 1 while the reactivity ratio of MMA, *r_MMA_*, is above 1. In the case of methacryloxypropyl-heptaisobutyl POSS, the reactivity ratios for POSS and MMA are 0.584 and 1.607, respectively. This means that MMA is preferentially incorporated into the copolymer and the amount of POSS introduced increases as the reaction progresses. The difference in the reactivity of the two comonomers can be explained by the significant difference in their molecular size and in the steric hindrance they exert [32]. Very recently, a higher reactivity of MMA and butyl methacrylate in copolymerization with methacryloxypropyl-heptaisobutyl POSS was also reported [33]. Interestingly, the opposite situation was indicated for the monomer pair, AdMA-MMA, with reactivity ratios of *r_AdMA_* = 1.25 and *r_MMA_* = 0.79 [27].

However, in the case of LM/1M-POSS and LM/TSM pairs, the situation may be different. Re-inspection of Figure 6 and Figure 8 indicates that the course of the kinetic curves is very similar in the steady state region for all formulations (the kinetic curves are almost parallel) and the possible compositional drift does not seem important. Therefore, based on the behavior of the kinetic curves, we can conclude that the dominant effect of the monomers with bulky substituents on the termination kinetics is rather the anchor effect.

#### 3.3.2. Photorheological Study

The second technique used to follow the polymerization kinetics was photorheology. The curing of the investigated formulations was monitored, with the rheometer registering the storage and loss modulus, *G’* and *G”*, respectively, as a function of the irradiation time at 40 °C (Figures S1 and S2). Because the polymers and copolymers are linear and their physical state can be described as viscous liquids [34], the moduli do not intersect (intersection corresponds to the gel point). Discussion of the photopolymerization kinetics is usually based on the evolution of the storage modulus [35,36,37] and the final values, *G’_f_*, of the storage modulus, *G’* (at the plateau region), are a measure of the final conversion [37]. For our systems, changes in *G’_f_* occurring as the modifiers’ content increases reflect both the effect of changes in the double bond conversions (chemical effect) and to a much greater extent the effect of the increasing amounts of pendant anchor groups (physical effect).

**Figure 9:**
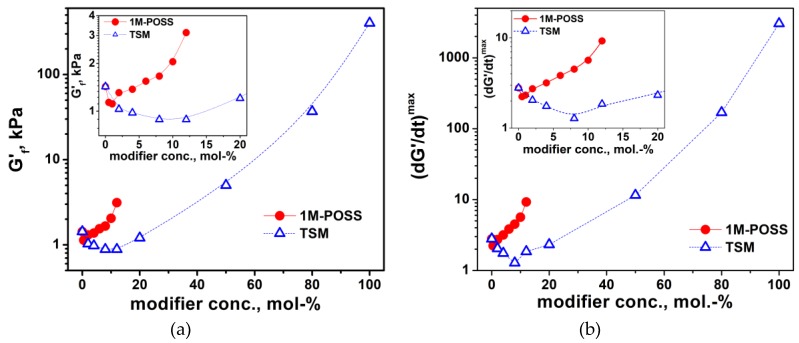
(**a**) Final storage modulus, *G_f_’*, and (**b**) the maximum value of *G’* derivative *(dG’/dt)^max^* as a function of the modifier content. Reaction temperature is 40 °C; the lines are eye guides.

The dependence of *G’_f_* on the modifier concentration is shown in Figure 9. The final values of the modulus are very low, which results from the physical state of the copolymers. These values are reduced at low concentrations of the modifiers (compared to poly-LM), which confirms their plasticizing effect, but increase drastically at higher concentrations mainly due to the stiffening effect of the anchor substituents. An analogous effect is shown by the final values of the complex viscosity, η*_f_ (Figure S3a). The changes in complex viscosity during the polymerization are shown in Figure S3b–d; the general picture of the obtained dependences is analogous to the changes in moduli. The modulus of neat poly-TSM is about 500 times higher than that of poly-LM.

However, the derivative of the modulus with respect to time, *dG’/dt*, corresponds to the polymerization rate, and its changes over time reflect changes in the reaction rate. Thus, the maximum value of the derivative, *(dG’/dt) ^max^*, corresponds to the maximum polymerization rate (at the inflection point of the curves in Figure S2). Figure 9b presents the *(dG’/dt) ^max^* dependence on the 1M-POSS and TSM contents. The plot of the function, (*dG’/dt) ^max^ = f (modifier conc.)*, exactly corresponds to the behavior of the *R_p_^max^* values determined by the DSC method (compared with Figure 7a). Due to the greater accuracy of the photorheological measurement, the minima on the plots are clearly visible at 1 and 8 wt % of 1M-POSS and TSM, respectively.

#### 3.3.3. Polymerization Rate Coefficients

Any change in the polymerization rate reflects changes in the polymerization rate coefficients. As mentioned earlier, the accelerating effect of the additives may be caused by a suppression of termination (reduction of *k_t_^b^*) and/or acceleration of the propagation (increase in *k_p_*), compared to Equation (1). The discussion presented above suggested the conclusion that the accelerating effect of 1M-POSS and TSM is associated with the anchor effect that inhibits termination. To find out whether our modifiers affect only the termination or also the propagation, the rate coefficients (in the form of *k_p_ × F* and *k_t_^b^ × F*) were calculated for the neat monomers (LM and TSM) and for compositions containing 10 mol % of 1M-POSS and 80 mol % of TSM. The compositions were selected to obtain large differences in the polymerization rates with respect to the neat LM. It is noteworthy that a similar increase in *R_p_^max^*, as with the addition of 10 mol % 1M-POSS, requires the addition of up to 50 mol % TSM.

**Figure 10:**
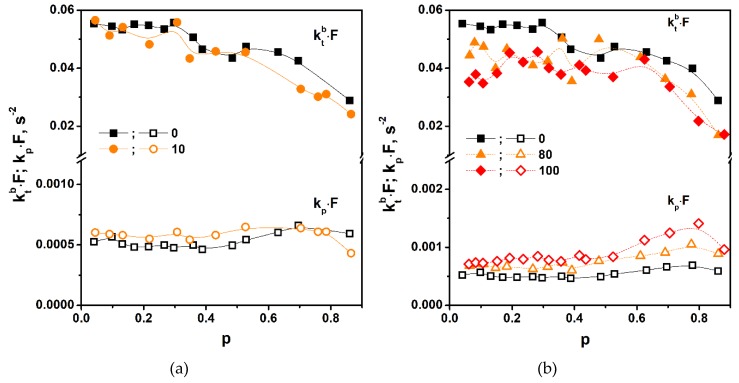
Parameters related to propagation (*k_p_⋅F*) and termination (*k_t_^b^⋅F*) rate coefficients as a function of the double bond conversion (**a**) LM/1M-POSS system and (**b**) the LM/TSM system. The numbers indicate the modifier content (mol %) in the mixture. Polymerization temperature: 40 °C. The lines are eye guides.

Parameters related to the rate coefficients of propagation, (*k_p_ × F*), and termination, (*k_t_^b^ × F*), as functions of the double bond conversion, p, are shown in Figure 10. These parameters were only to describe the tendencies of changes in the actual *k_p_* and *k_t_^b^* coefficients after adding modifiers with bulky substituents.

For the mixtures of the monomers, the calculated values were the resultants of the homopolymerization and copolymerization rate coefficients of the individual comonomers.

The *k_p_ × F* and *k_t_^b^ × F* parameters show a classical dependence on double bond conversion [20]. The *k_p_×F* values practically do not change to high conversions (propagation is not diffusion controlled); their slight increase at medium conversions (in the region of the gel effect) results from the deficiency of the model used for calculations. Regarding the *k_t_^b^ × F* parameter, at the steady state region (termination controlled by segmental diffusion), its values show a plateau up to conversions of about 0.3 ÷ 0.4. When the gel effect begins (termination becomes controlled by translational diffusion), *k_t_^b^ × F* starts to decrease with the increase in conversion. The *k_t_^b^* behavior in different reaction stages is better visible on the *k_t_^b^*/*k_p_* = *f*(*p*) dependence (Figure S4). Because the gel effect occurs at relatively high conversions, the reaction stage at which the termination is reaction-diffusion controlled is practically not observed; a second plateau corresponding to this stage is visible only at the highest conversions on the two curves in Figure S4 (from *p* ~ 0.7 ÷ 0.8). The practical lack of the reaction diffusion controlled termination region is probably associated with the fact that poly-LM is a highly viscous liquid even at room temperature.

Concerning the effect of the modifiers, it is clear that—as expected—they reduce the *k_t_^b^* coefficient. The reduction is moderate—for the LM/(1M-POSS 12 mol %) formulation by about 3.6% and in the case of LM/(TSM 80 mol %) by about 14%. The unexpected result is that *k_p_* of neat TSM is about 1.7 times higher than *k_p_* of LM. This clearly indicates that TSM reactivity is higher than that of LM. We can expect a similar result for the LM/1M-POSS pair. The *k_p_ × F* parameters in the two copolymerization processes are higher by 43% for TSM and by 26% for 1M-POSS compared to LM. Because the polymerization rate is inversely proportional to the square root of *k_t_^b^* and directly proportional to *k_p_*, the obtained results lead to the important conclusion that the accelerating effect of the modifiers (at concentrations higher than a few mol %) on LM polymerization results, to a greater extent, from the increase in the propagation rate rather than from suppression of termination.

An explanation of this result is difficult. Both anchor groups cause high steric hindrance, which should impede the access of the reactive group to the growing macroradical. In the published literature, the *k_t_^b^* coefficient of branched tert-butyl methacrylate (tert-BMA) has been reported to be lower than that of linear n-butyl methacrylate (n-BMA), as attributed to the effects of chain mobility hindered to a greater extent in tert-BMA than in n-BMA polymerization [38]. On the other hand, the *k_p_* values of methacrylates with cyclic ester groups (e.g., benzyl, cyclohexyl or isobornyl methacrylate) are clearly higher than *k_p_* of LM [39]. This is consistent with the results of the homopolymerization of the three monomers. It seems also that in our case the cross-propagation is not slower, because the modifiers enhance the polymerization rate. We can speculate that the apparently higher reactivity of the monomers with “anchor” groups results from the tendency of these groups to associate [18], which increases the monomer concentration in the close vicinity of the radical center. It is possible that in the case of the LM/modifier mixture, the physical interactions between the monomers lead to a similar effect, which may be another manifestation of the anchor effect (and its other interpretation)—anchoring the monomer with a long substituent around the POSS cage.

### 3.4. Copolymer Characterization

#### 3.4.1. GPC Analysis

**Figure 11:**
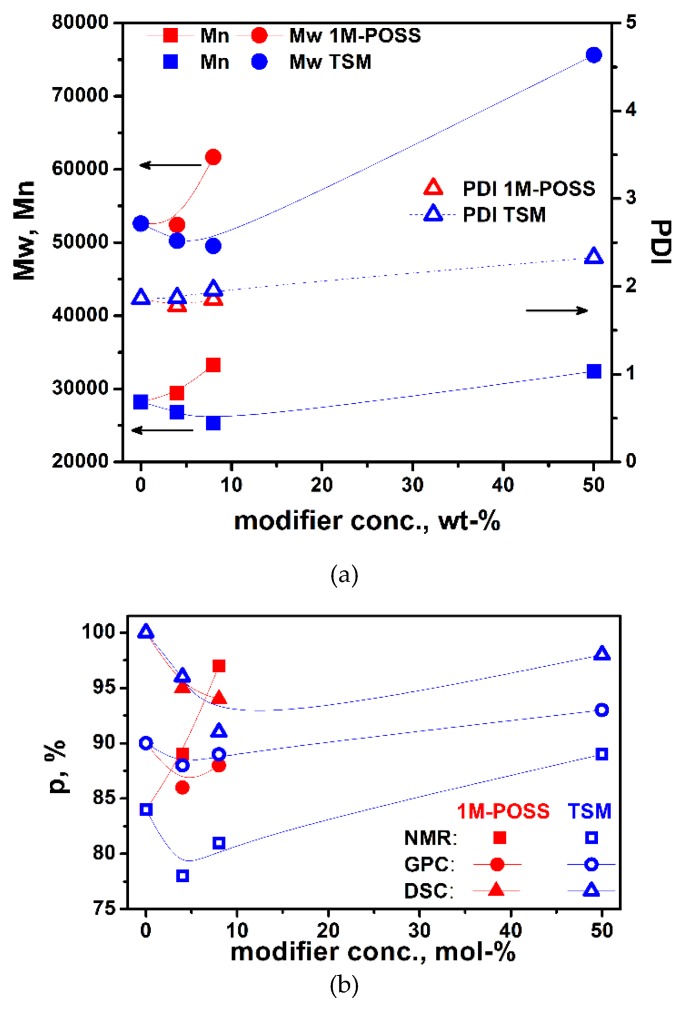
The dependence of (**a**) the copolymer molecular weight (determined by GPC) and (**b**) the copolymer yield (determined by various methods) on the modifier content. The lines are eye guides.

The results of GPC analysis (number average, *M_n_*, and weight average, *M_w_*, molecular weights, polydyspersity index, PDI, and fraction contents) of selected copolymerization products containing 4 and 8 mol % of 1M-POSS or 4, 8, and 50 mol % of TSM are given in Table S2. The dependence of the copolymer molecular weight (MW) and polydispersity index, PDI, on the modifier content is shown in Figure 11a. Interestingly, the plots show minima, analogously to the results obtained from the kinetic measurements. This seems to confirm the previous assumption that at low concentrations of the modifiers, the termination increases, while at higher contents, the anchor effect promotes chain elongation.

GPC analysis also enables the determination of the copolymer and monomer/oligomer residues’ contents. The results of the copolymer yield as a dependence of the modifier content are given in Figure 11b. The results are compared with the results obtained by other methods (DSC, NMR).

Again, the plots show the same feature: The existence of a minimum at low amounts of the introduced modifier. The difference in the *p^f^* values obtained from various methods results from the differences between the methods and parameters they measure directly. The decrease in the conversion with increasing modifier content is associated with the enhanced termination, while the increase in the conversion is with suppression of termination (and/or enhancement of propagation), which allows more monomer molecules to react.

#### 3.4.2. TEM Analysis

**Figure 12:**
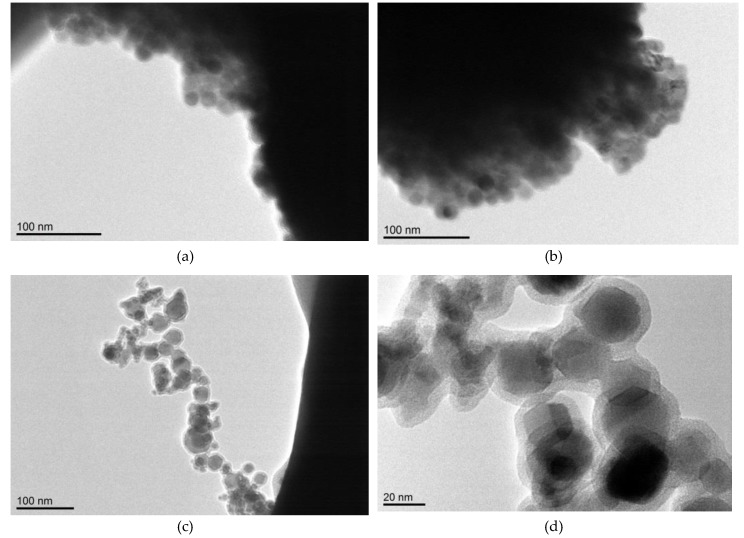
TEM images of (**a**) and (**b**) poly-LM/(1M-POSS 12); (**c**) and (**d**) poly-LM/(1M-POSS 0.5).

Figure 12 presents the TEM micrographs of copolymers containing 12 and 0.5 mol % of 1M-POSS.

TEM images presented in Figure 12a–b show the existence of spherical copolymer structures, which were probably formed by wrapping polymer chains around the aggregates of POSS cages. This can be better observed in Figure 12c–d. Inside spherical copolymer structures, the aggregates of POSS cages are clearly visible, forming, in many cases, hexagonal structures (octaalkyl-substituted cubic silsesquioxane nanoparticles form hexagonal, or equivalently rhombohedral, crystal structures [40]). This means that during the polymerization part of the pendant, POSS cages tend to form nanocrystals (on the order of 20 nm), and the polymer chains wrap around them. Due to the limited solubility of 1M-POSS in LM, such aggregation and wrapping (also of the monomer) probably begins at the early polymerization stages, which can positively affect the propagation rate.

### 3.5. Glass Transition Temperature, T_g_

**Figure 13:**
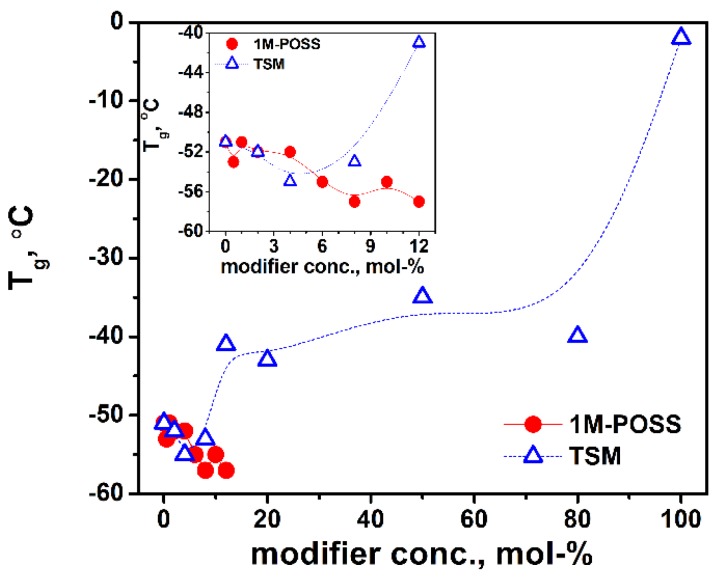
Glass transition temperature, *T_g_*, of LM/1M-POSS and LM/TSM copolymers as a function of the modifier content. The lines are eye guides.

The above-described effect of bulky substituents on the mobility of polymer chains should also be reflected in the values of glass temperature. Figure 13 shows *T_g_* plots against the modifier content. The minimum on the curve for the LM/TSM system appears again, whereas for the LM/1M-POSS system, only a drop in *T_g_* values is observed; however, we cannot rule out that with higher POSS contents, there would be an increase in *T_g_*.

It was indicated that POSS copolymerized with a base monomer can induce modifications to cooperative molecular motions typical of the glass transition, leading to retardation or acceleration of the dynamics. Therefore, the change in the glass transition temperature relative to the base polymer can be negative or positive and it is not correlated with the molecular weight [41]. In the case of MMA/1M-POSS copolymers (POSS concentrations up to 45 wt %), the significant negative deviations from *T_g_* of neat poly-MMA were observed in the entire range of comonomers ratios (as in our case). A similar effect was reported also for the copolymer of styrene and methacryloxystyrene-POSS functionalized with isobutyl groups [42]. Because the variation of the glass transition temperature in the random copolymers is the net result of several effects, free volume fraction, steric barrier, and POSS-polymeric segment interactions, the authors concluded that in their case, POSS-segment interactions are dominated by the internal plasticization (by isobutyl groups) and local free volume addition. Similarly, we can expect that in our case, the steric hindrance of the POSS cages that causes difficulties in the physical movements of the polymer chain is overcome by the flexibility of isobutyl groups. A monotonic decrease of the glass-transition temperature occurred also in the case of MMA copolymers with increasing amounts of methacrylcyclohexyl POSS and this was attributed to an increased free volume [43].

For LM/TSM copolymers, we observed both a negative and a positive change in *T_g_*, which confirms the effect of the branched siloxy-silane group on the mobility of the polymer chains, as discussed above (Section 3.3.1). This influence is analogous to that observed for the storage modulus and polymerization rate. A similar effect (decrease and increase in *T_g_*) for copolymers of styrene and methacryloxystyrene-POSS containing cyclohexyl vertex groups was described in [42]. In this case, intermolecular POSS-PS segment interactions were important for the *T_g_* values of the copolymers, with competition between the free volume and intermolecular interactions. We can assume a similar explanation for the behavior of our LM/TSM copolymers.

## 4. Conclusions

The effect of the anchoring group on the detailed polymerization kinetics was investigated by DSC and photorheology using photochemical initiation. The monomer with the bulky substituent was monomethacryloxy POSS (1M-POSS); for comparison, a methacrylate with a branched siloxy-silane group (TSM) was also applied. Both monomers were copolymerized with LM at various molar ratios.

The addition of the comonomers containing “anchor objects” had a dual effect on termination in LM/modifier copolymerization:

An increase of the molecular mobility by increasing the free volume fraction through the bulky substituent (and the plasticizing effect exerted by isobutyl groups in 1M-POSS), which results in an enhanced mobility of radical centers, leads to an acceleration of termination, a weakening of the gel effect, and a slowing down of the polymerization; these effects dominated at low concentrations of the modifiers.

Retardation and slowdown of molecular motions due to the “anchor effect” suppress the termination, leading to the acceleration of the polymerization; these effects dominated at higher modifier contents.

As an effect, the dependencies of *R_p_^max^, p^f^* (determined by NMR, GPC, and DSC), MW, and PDI on the modifier content showed the minima. The threshold concentration was higher for TSM due to the smaller “anchor group” and the lower viscosity of the TSM-containing formulations.

Determination of the propagation, *k_p_*, and termination, *k_t_^b^*, rate coefficients led to the conclusion that the accelerating effect of the modifiers on LM polymerization results both from the suppression of termination as well as from the increase in propagation The increase in *k_p_* led to an additional interpretation of the anchor effect: Anchoring the monomer with a long substituent (LM) around the POSS cage; this additionally enhanced the propagation by a local increase in the concentration of double bonds.

TEM images suggest that during polymerization, some of the pendant POSS cages formed nanocrystals (on the order of 20 nm), and polymer chains wrap around them. This could partially confirm the above supposition.

The *T_g_* behavior of TSM-containing copolymers was similar to the behavior of the polymerization rate (a minimum in the plot of *T_g_* vs. the modifier content), resulting from the influence of TSM on polymer chain mobility. In the case of 1M-POSS, only a plasticizing effect was observed.

In conclusion, we can say that the anchoring effect in the polymerization kinetics (for the monomers used in this work) affects the termination and propagation steps. The direction of this influence on termination depends on the concentration of the monomer containing the anchor object.

## Figures and Tables

**Table 1:** Investigated compositions.

LM/1M-POSS		LM/TSM	
1M-POSS Concentration		TSM Concentration	
mol %	wt %	mol %	wt %
0.0	0.0	0.0	0.0
0.5	1.8	2.0	3.3
1.0	3.6	4.0	6.5
2.0	7.0	8.0	12.6
4.0	13.4	12.0	18.5
6.0	19.1	20.0	29.4
8.0	24.4	50.0	62.4
10.0	29.2	80.0	86.9
12.0	33.6	100.0	100.0

## Supplementary Materials

The following are available online at https://www.mdpi.com/2073-4360/11/3/515/s1, Figure S1: (a) Storage modulus *G’* and (b) loss modulus *G”* as functions of irradiation time *t* at 40 °C for LM/1M-POSS system; Figure S2 (a, b) Storage modulus *G’* and (c, d) loss modulus *G”* as functions of irradiation time *t* at 40 °C for LM/TSM system; Figure S3: Final complex viscosity *ƞ*f* as a function of the modifier content (a) and complex viscosity as a function of the reaction time for (b) LM/1M-POSSsystem, (c) and (d) LM/TSM system; Figure S4: Dependence of the *k_t_^b^/k_p_* ratio on double bond conversion: (a) LM/1M-POSS system and (b) LM/TSM system; Table S1: Total monomer conversion determined from 1H NMR spectra and Table S2: Average molecular weights, PDI and fractions contents of the base polymer and copolymers.
