# Supplementary material for: Anchor Effect in Polymerization Kinetics: Case of Monofunctionalized POSS
Source: Polymers (Basel). 2019 Mar 19;11(3):515. doi: 10.3390/polym11030515 (PMC6473415; doi:10.3390/polym11030515)
Supplement: Supplementary file 1 [file polymers-11-00515-s001.pdf]

Supplementary Materials

Anchor Effect in Polymerization Kinetics: Case of Monofunctionalized POSS

Agnieszka Marcinkowska[1*], Dawid Przadka[1], Beata Dudziec[2], Katarzyna Szczesniak[3], Ewa Andrzejewska[1*]

[1] Poznan University of Technology, Faculty of Chemical Technology, Poznan, Berdychowo 4, 60-965 Poznan, Poland; dawprza@wp.pl

[2] Adam Mickiewicz University in Poznan, Faculty of Chemistry and Centre for Advanced Technologies, Umultowska 89B and C, 61-614 Poznan, Poland; beata.dudziec@gmail.com

[3] NanoBioMedical Center, Adam Mickiewicz University, Umultowska 85, 61-614 Poznan, Poland; k.szczesniak@amu.edu.pl

[*] Correspondence: agnieszka.marcinkowska@put.poznan.pl, ewa.andrzejewska@put.poznan.pl; Tel.: +48-61-665-36-05 (A.M.), +48-61-665-36-37 (E.A.)

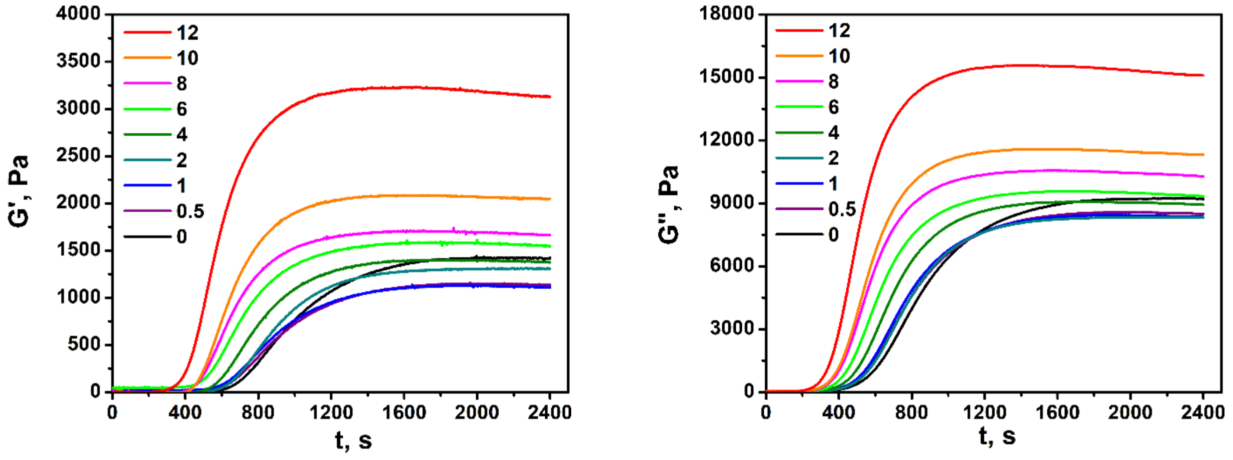

(a) (b)

Figure S1. (a) Storage modulus *G′* and (b) loss modulus *G″* as functions of irradiation time *t* at 40°C for LM/1M-POSS system. The numbers indicate 1M-POSS content (mol-%) in the composition

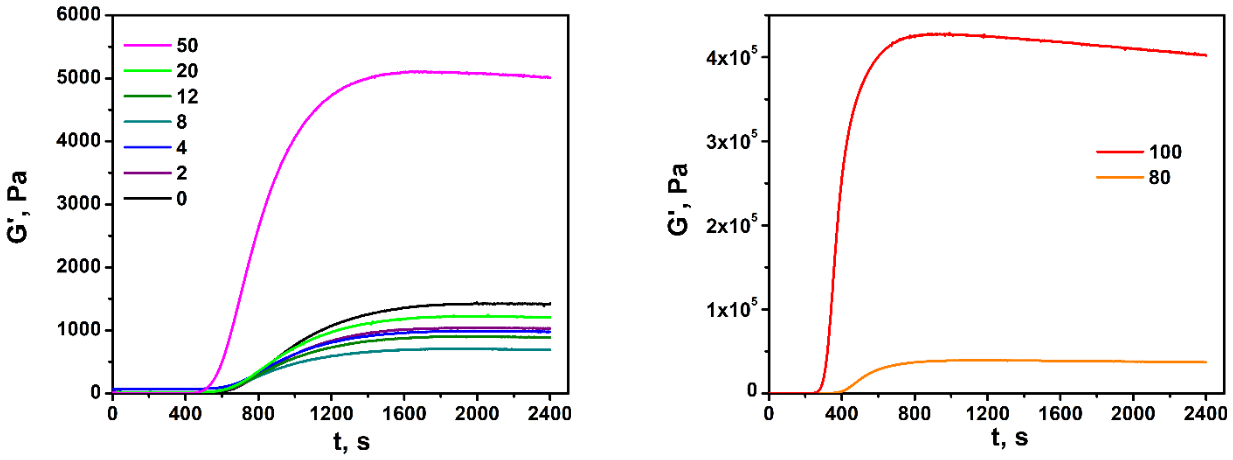

(a) (b)

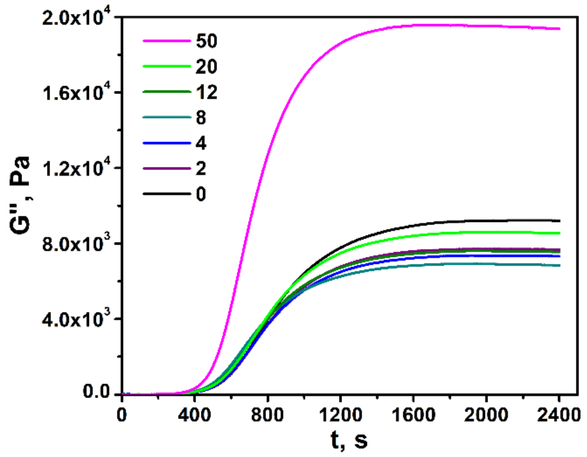

(c)

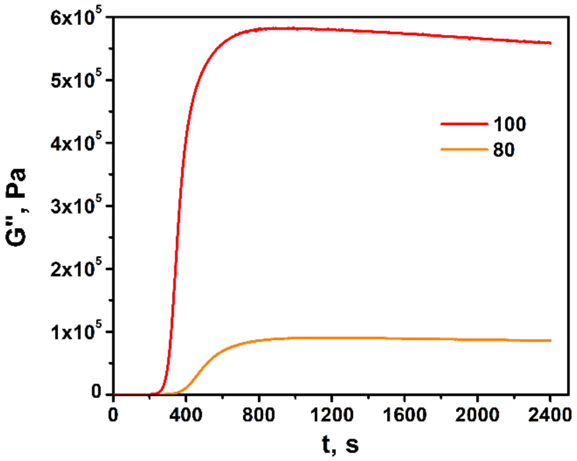

(d)

Figure S2. (a, b) Storage modulus G' and (c, d) loss modulus G'' as functions of irradiation time t at 40°C for LM/TSM system. The numbers indicate TSM content (mol-%) in the composition

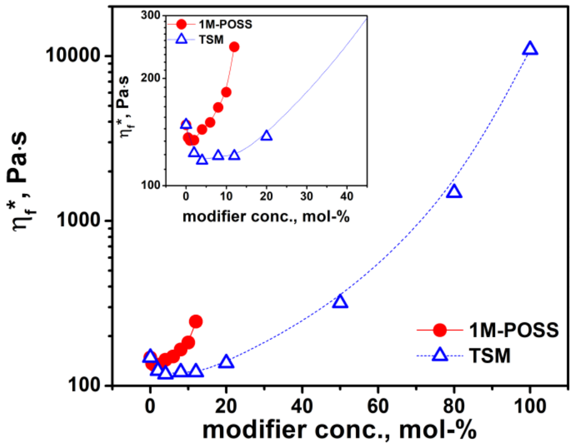

(a)

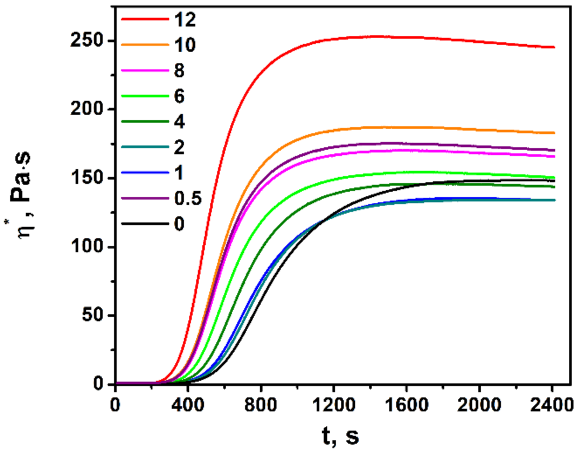

(b)

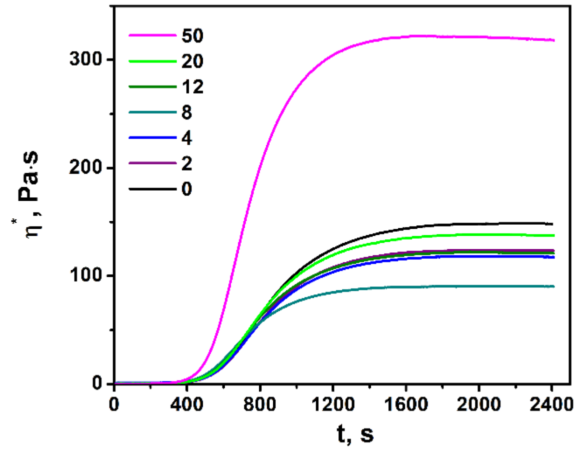

(c)

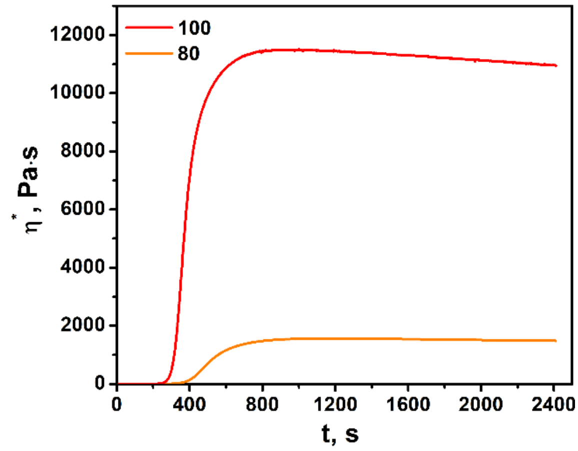

(d)

Figure S3. Final complex viscosity η^*_f as a function of the modifier content (a) and complex viscosity as a function of the reaction time for (b) LM/1M-POSS system, (c) and (d) LM/TSM system. The numbers indicate modifier content (mol-%) in the composition. The lines are guides for eyes.

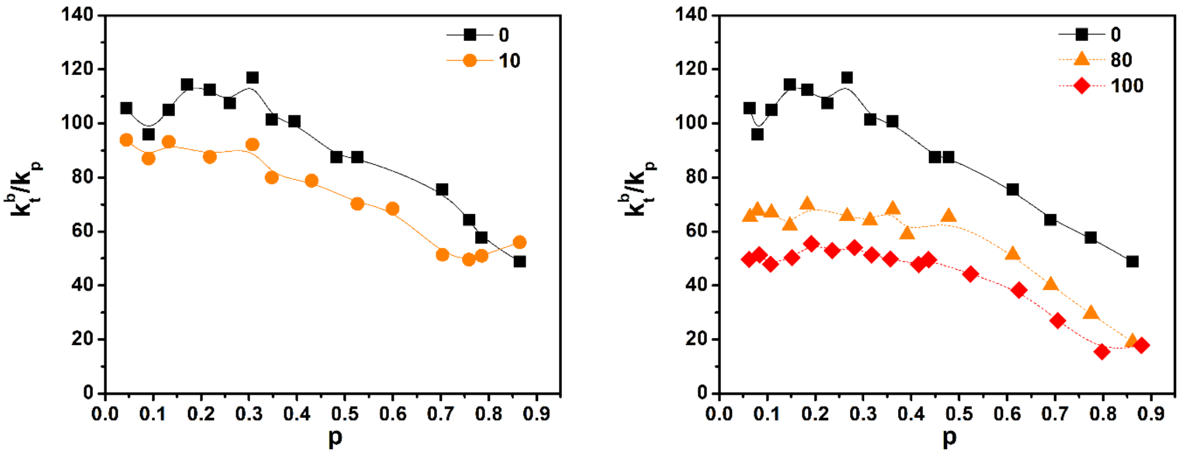

(a) (b)

Figure S4. Dependence of the k_t^b/k_p ratio on double bond conversion: (a) LM/1M-POSS system and (b) LM/TSM system. The numbers indicate the modifier content (mol-%) in the mixture. Polymerization temperature: 40°C. The lines are guides for eyes.

Table S1. Total monomer conversion determined from ^{1}H NMR spectra.

**Total monomer conversion, %***					
poly-LM	poly-LM/ (1M-POSS 4)	poly-LM/ (1M-POSS 8)	poly-LM/ (TSM 4)	poly-LM/ (TSM 8)	poly-LM/ (TSM 50)
84	89	97	78	81	89

** The numbers in the copolymer names indicate the modifier content in mol-%.*

Table S2. Average molecular weights, PDI and fractions contents of the base polymer and copolymers.

	**Mn, g·mol^{-1}**	**Mw, g·mol^{-1}**	**PDI**	**Fraction content, %**
poly-LM	28243	52594	1.86	90.3
	577	644	1.12	9.7
poly-LM/(POSS 4)	29422	52429	1.78	85.8
	1981	2315	1.17	5.2
	569	623	1.09	9.0
poly-LM/(POSS 8)	33308	61675	1.85	88.1
	2016	2383	1.18	7.9
	601	639	1.06	3.5
	198	209	1.06	0.5
poly-LM/(TSM 4)	26833	50254	1.87	87.7
	600	662	1.10	12.3
poly-LM/(TSM 8)	25282	49550	1.96	89.0
	574	662	1.15	11.0
poly-LM/(TSM 50)	32401	75645	2.33	93.4
	702	799	1.14	6.4
	189	197	1.04	0.2
